# Prediction of Causative Genes in Inherited Retinal Disorders from Spectral-Domain Optical Coherence Tomography Utilizing Deep Learning Techniques

**DOI:** 10.1155/2019/1691064

**Published:** 2019-04-09

**Authors:** Yu Fujinami-Yokokawa, Nikolas Pontikos, Lizhu Yang, Kazushige Tsunoda, Kazutoshi Yoshitake, Takeshi Iwata, Hiroaki Miyata, Kaoru Fujinami, on behalf of Japan Eye Genetics Consortium

**Affiliations:** ^1^Laboratory of Visual Physiology, Division of Vision Research, National Institute of Sensory Organs, National Hospital Organization Tokyo Medical Center, Tokyo 152-8902, Japan; ^2^Graduate School of Health Management, Keio University, Tokyo 160-0016, Japan; ^3^UCL Institute of Ophthalmology, London EC1V 9EL, UK; ^4^Moorfields Eye Hospital, London EC1V 2PD, UK; ^5^Peking Union Medical College Hospital, Peking Union Medical College and Chinese Academy of Medical Sciences, Beijing 100006, China; ^6^Department of Ophthalmology, Keio University of Medicine, Tokyo 160-0016, Japan; ^7^Division of Molecular and Cellular Biology, National Institute of Sensory Organs, National Tokyo Medical Center, Tokyo 152-8902, Japan; ^8^Department of Health Policy and Management, School of Medicine, Keio University, Tokyo 160-8582, Japan

## Abstract

**Purpose:**

To illustrate a data-driven deep learning approach to predicting the gene responsible for the inherited retinal disorder (IRD) in macular dystrophy caused by *ABCA4* and *RP1L1* gene aberration in comparison with retinitis pigmentosa caused by *EYS* gene aberration and normal subjects.

**Methods:**

Seventy-five subjects with IRD or no ocular diseases have been ascertained from the database of Japan Eye Genetics Consortium; 10 *ABCA4* retinopathy, 20 *RP1L1* retinopathy, 28 *EYS* retinopathy, and 17 normal patients/subjects. Horizontal/vertical cross-sectional scans of optical coherence tomography (SD-OCT) at the central fovea were cropped/adjusted to a resolution of 400 pixels/inch with a size of 750 × 500 pix^2^ for learning. Subjects were randomly split following a 3 : 1 ratio into training and test sets. The commercially available learning tool, Medic mind was applied to this four-class classification program. The classification accuracy, sensitivity, and specificity were calculated during the learning process. This process was repeated four times with random assignment to training and test sets to control for selection bias. For each training/testing process, the classification accuracy was calculated per gene category.

**Results:**

A total of 178 images from 75 subjects were included in this study. The mean training accuracy was 98.5%, ranging from 90.6 to 100.0. The mean overall test accuracy was 90.9% (82.0–97.6). The mean test accuracy per gene category was 100% for *ABCA4*, 78.0% for *RP1L1*, 89.8% for *EYS*, and 93.4% for Normal. Test accuracy of *RP1L1* and *EYS* was not high relative to the training accuracy which suggests overfitting.

**Conclusion:**

This study highlighted a novel application of deep neural networks in the prediction of the causative gene in IRD retinopathies from SD-OCT, with a high prediction accuracy. It is anticipated that deep neural networks will be integrated into general screening to support clinical/genetic diagnosis, as well as enrich the clinical education.

## 1. Introduction

Inherited retinal disorder (IRD) that has been lacking effective treatment is a leading cause of blindness in developed countries [1–5], affecting around 1 in 3000 people worldwide. The successful identification of the causative genes for retinal dystrophies with next generation sequencing technologies has increased the number of associated genes and disease-causing variants; to date, over 250 genes and 300 genes and loci have been identified (RetNet: https://sph.uth.edu/retnet/sum-dis.htm). Emerging treatment approaches, such as gene replacement therapy, pharmacological agents, regenerative therapies, retina prosthesis, and others, have increased the therapeutic potential for IRDs [6, 7].

Recently, large cohort studies have provided powerful information to determine clinical manifestations of IRD, such as fundus appearance and morphological findings, and characteristic morphological features caused by specific genes in macular dystrophy are well established [8–14]. For instance, typical cases with *ABCA4* retinopathy show disruption of photoreceptor layers with thinned sensory retina at the macula, and cases with *RP1L1* retinopathy demonstrate blurring of photoreceptor ellipsoid zone (EZ) and loss of photoreceptor interdigitation zone (IZ) [8, 11, 15–22].

On the contrary, it has been challenging to make a diagnosis at general ophthalmology clinics without IRD specialists since detailed and specific phenotypic assessment is unavailable because of the limited information due to the rarity of IRD. Recently, applications of artificial intelligence (AI) including deep neural network have been increasingly developed for screening/predicting several common retinal diseases from fundus images or spectral-domain optical coherence tomographic (SD-OCT) images [23–28]. However, such characterization/classification using automatic image analysis methods has not been developed in orphan retinal diseases.

The purpose of our study was to illustrate a data-driven deep learning approach to predict the causative genes of IRD in macular dystrophy caused by *ABCA4* and *RP1L1* in comparison with retinitis pigmentosa caused by *EYS* gene aberration and normal subjects.

## 2. Materials and Methods

The protocol of this study adhered to the tenets of the Declaration of Helsinki and was approved by the Ethics Committee of the participating institutions: National Institute of Sensory Organs (NISO), National Hospital Organization, and Tokyo Medical Center (Reference number: R15-037). A signed informed consent was obtained from all patients.

For the purpose of this study, two most prevalent genes for macular dystrophies and one most prevalent gene for retinitis pigmentosa were selected from the dataset of Japan Eye Genetics Consortium (JEGC): proportion of *ABCA4* retinopathy, *RP1L1* retinopathy, and *EYS* retinopathy were 5.5%, 8.5%, and 15.9%, respectively.

In total, 75 patients/subjects with molecularly confirmed IRD or no ocular diseases have been ascertained from the JEGC database [11]: 10 patients with *ABCA4* retinopathy, 20 patients with *RP1L1* retinopathy, 28 with *EYS* retinopathy, and 17 normal subjects. SD-OCT images are obtained with three OCT devices (Cirrus HD-OCT; Carl Zeiss Meditec, Dublin, CA, USA, Spectralis OCT; Heidelberg Engineering, Franklin, MA, USA; Swept Source OCT DRI OCT-1 Atlantis, Topcon Corporation, Tokyo, Japan).

**Figure 1:**
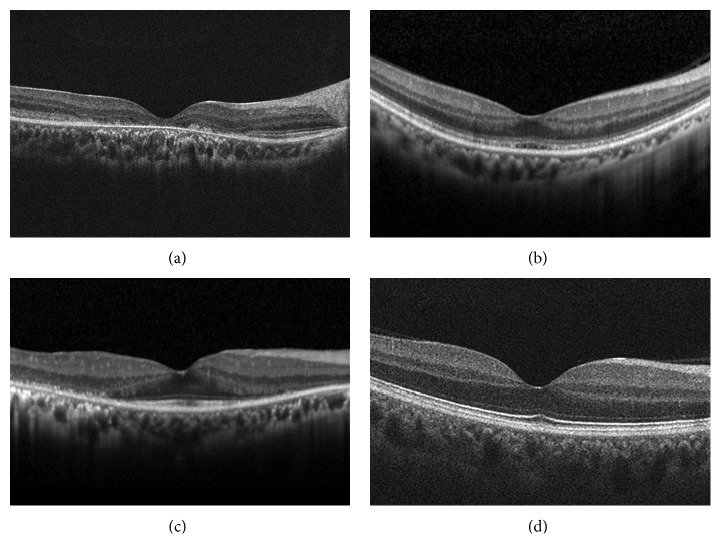
Spectral-domain optical coherence tomographic (SD-OCT) images of four categories in prediction of causative genes in inherited retinal disorders. For the purpose of this study, two most prevalent genes (*ABCA4*, *PR1L1*) for macular dystrophies and one most prevalent gene (*EYS*) for retinitis pigmentosa were selected from the dataset of Japan Eye Genetics Consortium. Characteristic morphological features are demonstrated in each spectral-domain optical coherence tomographic (SD-OCT) image. ABCA4 : disruption of photoreceptor layers with thinned sensory retina at the macula. RP1L1 : blurring of photoreceptor ellipsoid zone and loss of photoreceptor interdigitation zone at the macula. EYS : disruption of photoreceptor layers with thinned sensory retina at the paramacular with relatively preserved structure at the macula. Normal: normal retinal structures.

Horizontal and vertical cross-sectional scans of SD-OCT at the central fovea of both eyes were cropped and adjusted to a spatial resolution of 400 pixels/inch with a size of 750 × 500 pix^2^ for deep learning. Four gene categories were defined based on the clinical and genetic diagnosis; *ABCA4*, *RP1L1*, *EYS*, and Normal (Figure 1). After preparation of SD-OCT images for four gene categories, patients/subjects were randomly split following a 3 : 1 ratio into training and test sets.

The commercially available deep learning web tool, Medic Mind (https://www.medicmind.tech/), was applied to this four-class classification program [29]. The classification accuracy, sensitivity, and specificity were calculated during the learning process. The process was repeated four times with random assignment of subjects to training and test sets to control for selection bias, given the relatively small sample size. For each training/testing process, the classification accuracy was calculated per gene category.

## 3. Results and Discussion

A total of 178 images from 75 patients/subjects with IRD/Normal were included in this study. The detailed training and test results of deep learning performance in prediction of causative genes in IRD are presented in Table 1.

The mean training accuracy of the four repeated experiments was 96.9% and ranged from 90.6% to 100.0%. The mean sensitivity was 100% for ABCA4, 88.1 (66.7–100%) for RP1L1, 97.7 % (90.9–100%) % for EYS, and 100% for Normal. Sufficient training accuracy over 66.7% was obtained. The mean test accuracy of the four repeated experiments was 90.3%, ranging from 82.0% to 97.6%. The test accuracy per gene category was 100% for *ABCA4* and ranged from 66.7 to 87.5% for *RP1L1*, 82.4 to 100% for *EYS*, and 73.7 to 100% for Normal according to the four repeated experiments.

**Figure 2:**
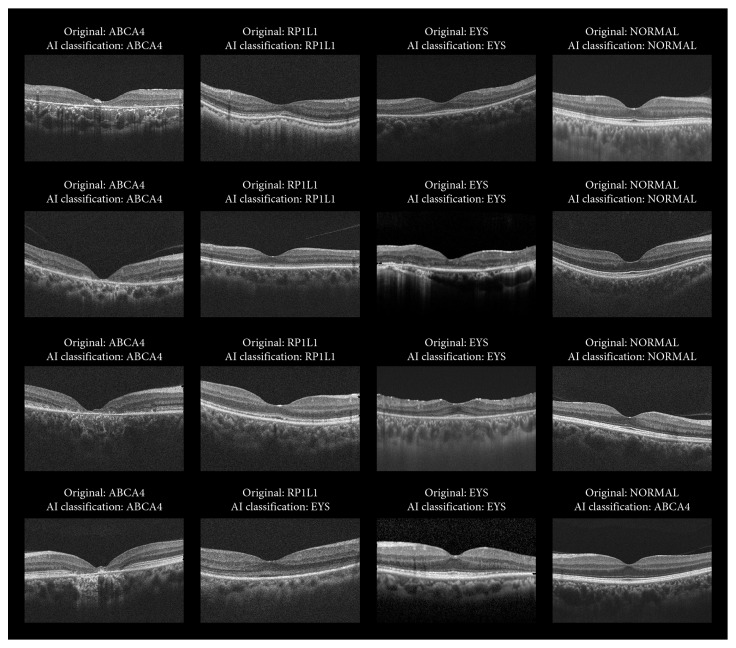
Test results of deep learning in 4 representative cases with original diagnosis of ABCA4, RP1L1, EYS, and Normal. A data-driven deep learning approach to predicting the gene causing the inherited retinal disorders in macular dystrophy caused by *ABCA4* and *RP1L1* in comparison with retinitis pigmentosa caused by *EYS* and normal subjects was performed with Medic Mind (URL: https://www.medicmind.tech/). The mean overall test accuracy was 90.9% (82.0–97.6). The test accuracy per gene category was 100% for *ABCA4*, 78.0% (66.7–87.5) for *RP1L1*, 89.8% (82.4–100) for *EYS*, and 93.4% (73.7–100) for Normal. AI = artificial intelligence.

The test accuracy of ABCA4 and Normal was considerably higher than that of RP1L1 and EYS, which suggests overfitting. Four (10.5%) out of 38 with an original diagnosis of RP1L1 were classified as EYS, two (5.3%) with RP1L1 were classified as Normal, and one (2.6%) with original RP1L1 was classified as ABCA4 (Figure 2). Two (3.6%) out of 56 with an original diagnosis of EYS were classified as ABCA4, two (3.6%) with original EYS were classified as RP1L1, and two (3.6%) with EYS were classified as Normal.

The potential efficacy of the automatic screening/diagnostic system of IRD from SD-OCT has been illustrated. To the best of our knowledge, this is the first report of utilizing deep learning technology in retinal orphan disorders.

Since IRD exhibits strong gene-characteristic morphological features which are less influenced by environmental factors, these present an ideal application of AI to assist medical diagnosis. Furthermore, in general, deducing IRD is not hard at general ophthalmology clinics, as the family history, specific chief complains, and symmetric findings are unique; complex diagnosis, therefore, with such demographic information and AI-guided assessment for SD-OCT could assist properly identifying patients with IRD.

Application of AI in common diseases such as diabetic retinopathy and age-related macular degeneration manifests huge impacts/effects on improvement of medical care and saving the cost. This study further supports the extended utility of AI in relatively common IRD (covering 30% of total IRD), which has a huge impact not only on ocular diseases but also on other orphan disorders.

Interestingly, the predicted classification of *ABCA4* and classification of Normal were highly accurate in this study, while there were still some difficulties in classifying *RP1L1* and *EYS* retinopathies. This may be because the characteristic features of *RP1L1* retinopathy with blurring of EZ and loss of IZ are hard to be detected from image analysis, and the disrupted zone of some *EYS* retinopathy might lie outside of the observed field in SD-OCT images.

There are several limitations in this study. The cohort size of this study was relatively small both in control and affected groups, and target ethnicity was only Japanese, in which larger cohort studies with data from various ethnicities could expand the potential utility of our approach. In our study, comparison analysis of the variation in diagnoses by human and AI prediction was not performed due to the limited data resources, which could delineate the usefulness and weakness of our approach. There are limitations to AI classifiers which are trained to only distinguish between a finite number of predefined classes for which we have sufficient training data. However, as we know, there are over 300 genes that can cause IRD; therefore, the four-class classifier we have developed here cannot be usefully applied in practice to predict a gene from an SD-OCT of a patient with a novel gene diagnosis. It would however be expected that our algorithm would return probabilities close to 25% (1/4) if presented with an SD-OCT which does not fit any of the four classes. Instead, another more clinically applicable approach may be to train several one-or-rest classifiers to distinguish one gene from all others. These could then be applied successively to an SD-OCT to assess which gene diagnosis is the best fitting. However, this would require training several classifiers and would require a sufficient number of cases for each gene classifier, which might not be possible for the rarer IRDs. There is also a current interpretability limitation to AI classifiers which use deep learning. This field remains an active research area, and we intend to explore approaches such as saliency and occlusion maps to highlight what parts of an SD-OCT are important to the classification decision.

Our approach could be extended to other retinopathy-associated genes/loci, of which they are over 300. The challenge lies in obtaining sufficiently large training datasets for the rarest forms of IRD and in distinguishing similar morphological changes within the same mechanism group/cascade (e.g., generalized rod dysfunction, generalized cone dysfunction, and confined macular dysfunction). Further comprehensive studies in larger cohorts, in combination with other phenotypic modalities such as fundus appearance, fluorescein angiography, visual fields, and electrophysiological findings, and imaging performed at different time points in the progression of disease should improve the performance of these prediction algorithms.

## 4. Conclusions

This study highlighted a novel application of deep neural networks in the prediction of the major causative genes (30%) in IRD retinopathies from SD-OCT, with a mean prediction accuracy of 90%. It is anticipated that deep neural networks will be integrated into general screening to support clinical diagnosis, suggest a causative gene to guide genetic screening, as well as enrich clinical education of orphan retinal disease.

## Figures and Tables

**Table 1:** Summary of deep learning performance in prediction of causative genes in inherited retinal disorders.

Experiment 1.		Training results				Test 1.		Test results						Total number of images included in this study	
		Number of images	Sensitivity (%)	Specificity (%)	Accuracy (%)			Original category of genetic diagnosis					Accuracy (%)		
								ABCA4	RP1L1	EYS	Normal	Total			
Original classification of genetic diagnosis	ABCA4	15	100	100	—	Predicted category of genetic diagnosis	ABCA4	4	1	—	—	5	100	ABCA4	19
	RP1L1	29	85.7	100	—		RP1L1	—	7	—	—	7	87.5	RP1L1	37
	EYS	43	100	95	—		EYS	—	—	14	—	14	100	EYS	57
	Normal	49	100	100	—		Normal	—	—	—	16	16	100	Normal	65
	Total	136	—	—	96.9		Total	4	8	14	16	42	97.6	Total	178

Experiment 2.		Training results				Test 2.		Test results						Total number of images included in this study	
		Number of images	Sensitivity (%)	Specificity (%)	Accuracy (%)			Original category of genetic diagnosis					Accuracy (%)		
								ABCA4	RP1L1	EYS	Normal	Total			

Original classification of genetic diagnosis	ABCA4	13	100	100	—	Predicted category of genetic diagnosis	ABCA4	6	2	1	—	9	100	ABCA4	19
	RP1L1	26	100	100	—		RP1L1	—	9	1	2	12	82	RP1L1	37
	EYS	43	100	100	—		EYS	—	—	12	3	15	85.7	EYS	57
	Normal	46	100	100	—		Normal	—	—	——	14	14	73.7	Normal	65
	Total	128	—	—	100		Total	6	11	14	19	50	82	Total	178

Experiment 3.		Training results				Test 3.		Test results						Total number of images included in this study	
		Number of images	Sensitivity (%)	Specificity (%)	Accuracy (%)			Original category of genetic diagnosis					Accuracy (%)		
								ABCA4	RP1L1	EYS	Normal	Total			

Original classification of genetic diagnosis	ABCA4	15	100	96.6	—	Predicted category of genetic diagnosis	ABCA4	4	—	1	—	5	100	ABCA4	19
	RP1L1	31	66.7	100	—		RP1L1	—	4	—	—	4	66.7	RP1L1	37
	EYS	45	90.9	90.5	—		EYS	—	2	10	—	12	90.9	EYS	57
	Normal	49	100	100	—		Normal	—	—	—	16	16	100	Normal	65
	Total	140	—	—	90.6		Total	4	6	11	16	37	91.9	Total	178

Experiment 4.		Training results				Test 4.		Test results					Accuracy (%)	Total number of images included in this study	
		Number of images	Sensitivity (%)	Specificity (%)	Accuracy (%)			Original category of genetic diagnosis							
								ABCA4	RP1L1	EYS	Normal	Total			

Original classification of genetic diagnosis	ABCA4	14	100	100	—	Predicted category of genetic diagnosis	ABCA4	5	—	—	—	5	100	ABCA4	19
	RP1L1	25	100	100	—		RP1L1	—	10	1	—	11	76.9	RP1L1	37
	EYS	40	100	100	—		EYS	—	—	14	—	14	82.4	EYS	57
	Normal	51	100	100	—		Normal	—	2	2	14	18	100	Normal	65
	Total	130	—	—	100		Total	5	13	17	14	49	89.8	Total	178

In total, 75 subjects with molecularly confirmed inherited retinal disorders or no ocular diseases have been ascertained: 10 with *ABCA4* retinopathy, 20 patients with *RP1L1* retinopathy, 28 with EYS retinopathy, and 17 normal subjects. After preparation of spectral-domain optical coherence tomographic (SD-OCT) images for four gene categories, subjects were randomly split following a 3 : 1 ratio into training and test sets. The commercially available deep learning web tool, Medic Mind, was applied to this four-class classification problem. The classification accuracy, sensitivity, and specificity were calculated during the learning process, and the process was repeated four times with randomly assigned training/test sets to control for selection bias. For each training/testing process, the classification accuracy was calculated per gene category.

## Data Availability

Raw data were generated in the Japan Eye Genetics Consortium database and obtained with permission of the Japan Eye Genetics Consortium at https://niso.kankakuki.go.jp/opkarte/. Derived data supporting the findings of this study are available from the corresponding author on request.
